# Beware of *N*-Benzoyloxybenzamides

**DOI:** 10.3390/molecules29215143

**Published:** 2024-10-31

**Authors:** Jonathan Cubitt, Mari Davies, Ross Riseley, Gabrielle Evans, Sian E. Gardiner, Benson M. Kariuki, Simon E. Ward, Emyr Lloyd-Evans, Helen Waller-Evans, D. Heulyn Jones

**Affiliations:** 1 Medicines Discovery Institute, Cardiff University, Main Building, Park Place, Cardiff CF10 3AT, UK; cubittjm@gmail.com (J.C.); daviesmf4@cardiff.ac.uk (M.D.); riseleyr@cardiff.ac.uk (R.R.); evansg62@cardiff.ac.uk (G.E.); gardiners1@cardiff.ac.uk (S.E.G.); kariukib@cardiff.ac.uk (B.M.K.); wards10@cardiff.ac.uk (S.E.W.); lloyd-evanse@cardiff.ac.uk (E.L.-E.); 2 Chemistry Department, Cardiff University, Cardiff CF10 3AT, UK

**Keywords:** *N*-benzoyloxybenzamides, acid ceramidase, PAINS

## Abstract

Following a High-Throughput Screening campaign to discover inhibitors of acid ceramidase, we report the novel and extremely potent covalent inhibitor, **1**. Following resynthesis and stability monitoring, we discovered that **1** is chemically unstable and reacts with DMSO at room temperature. This mode of decomposition is likely general for this class of compound, and we urge caution for their use in drug discovery research.

## 1. Introduction

**Figure 1:**
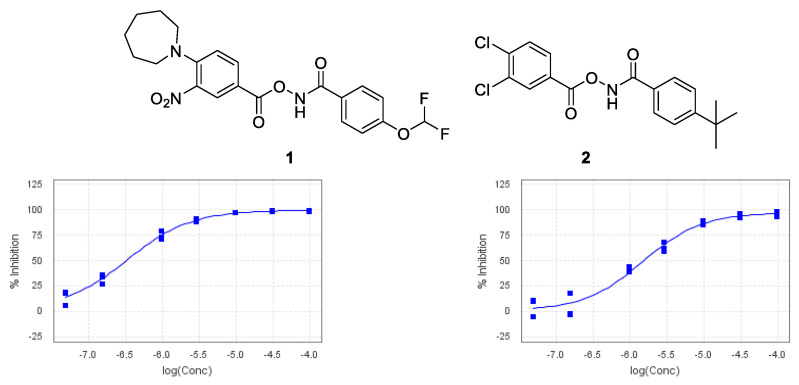
Chemical structures and HTS IC_50_ curves.

Despite continuing advances in structure-driven research, High-Throughput Screening (HTS) is still a valuable resource to find novel chemical starting points in drug discovery [1]. Recently, the Medicines Discovery Institute initiated an HTS looking for small-molecule inhibitors of acid ceramidase, a lysosomal enzyme. Using a modified reported acid ceramidase assay [2,3], a number of potential chemical starting points were identified, the most potent of which was **1**, with an IC_50_ value of 340 nM with a structurally related compound also showing moderate inhibition (Figure 1). Samples of **1** and **2** were subsequently tested in our in-house cell lysate acid ceramidase inhibition assay, showing an increased activity of 11 nM and 237 nM, respectively. This substantial increase in potency can be explained by the fact that to function properly, acid ceramidase requires a co-factor present in cells which was not present in the original HTS assay, Saposin D [4].

**Figure 2:**
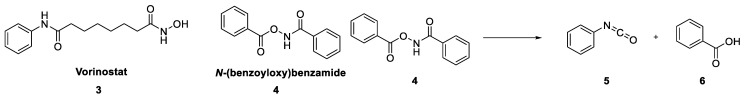
Examples of biologically active hydroxamic acids and the Lossen rearrangement reaction.

Although we could not find any direct mention of *N*-(benzoyloxy)benzamides as Pan-Assay Interference Compounds (PAINS), we were extremely sceptical of **1** as a useful chemical starting point for our project due to the unusual and potentially reactive functionality at the centre of the molecule. There are instances of hydroxamic acids in clinical use, such as the histone deacetylase inhibitor Vorinostat (**3**) [5,6], whose mechanism of action was linked to the chelation of Zn^2+^. There were also several reports of the biological activity of *N*-(benzoyloxy)benzamide (**4**) and close analogues [7,8,9]. Worryingly, linked to these reports was the mention of the general mutagenic properties of many hydroxamic acids [10], believed to occur through a Lossen rearrangement reaction to form the highly reactive isocyanate product (Figure 2) [11].

## 2. Results and Discussion

Before committing to a resynthesis of **1**, we decided to rule out products of the Lossen rearrangement as potential culprits for the observed biological activity. Compound libraries are often kept for a long period of time and undergo several freeze/thaw cycles, where **1** may have undergone Lossen rearrangement. LCMS QC analysis of the supplied sample of **1** suggested a purity of 82% by UV. It was still possible, however, that either a small amount had undergone the rearrangement to form an extremely potent product, or that rearrangement had occurred under the conditions of our assay.

**Scheme 1:**
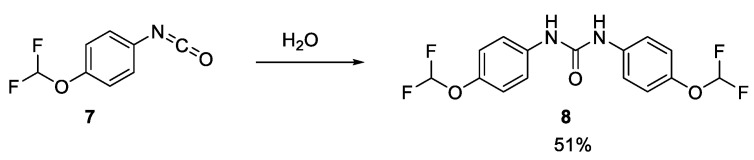
Hydrolysis of isocyanate **7**.

To verify this, a sample of the rearrangement product, isocyanate **7**, was purchased and tested in our cell lysate assay. **7** was also hydrolysed with water, giving **8**, which was also tested in our cell lysate assay (Scheme 1) [12]. Both samples only inhibited acid ceramidase very weakly (IC_50_ value of around 100 µM). LCMS analysis of the isocyanate DMSO stock sample indicated that **7** had already fully hydrolysed to **8**, which explains the near-identical level of inhibition. It was still possible that the other product of the Lossen rearrangement, the carboxylic acid **12,** was the active species. However, since this acid was not commercially available and was an intermediate in the resynthesis of **1**, the decision was made to commit to the resynthesis of the hit compound.

**Scheme 2:**
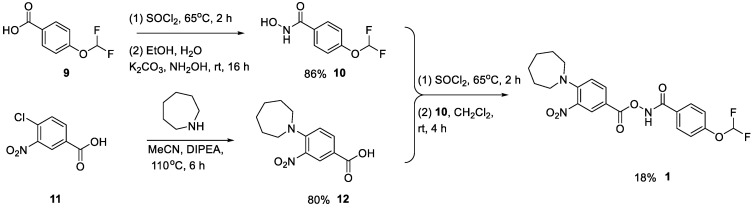
Synthesis of hit compound **1**.

Formation of the hydroxamic acid via the acid chloride proceeded smoothly, giving **10** in 86% yield. The carboxylic acid **12** was accessed through an SNAr reaction of azepane onto 4-chloro-3-nitrobrenzoic acid. Conversion to the product was slow using conventional heating methods. Prolonged heating in a Biotage microwave with excess amine drove the reaction to completion, giving **12** in 80% yield. Since **12** was the other possible product from the Lossen rearrangement, it was tested in our cell lysate inhibition assay but was inactive. The final step proceeded smoothly, again via an acid chloride. Purification by automated reverse-phase chromatography gave the target compound, **1**, in 18% yield (Scheme 2).

With a freshly synthesised sample in hand, **1** was tested in both our cell lysate and purified protein acid ceramidase inhibition assays. **1** was confirmed as an extremely potent inhibitor of acid ceramidase, with an IC_50_ value of 3.2 nM and 163 nM in the respective assays. Despite the apparent impressive potency of this compound, we continued to have concerns over using **1** as a chemical starting point.

**Figure 3:**
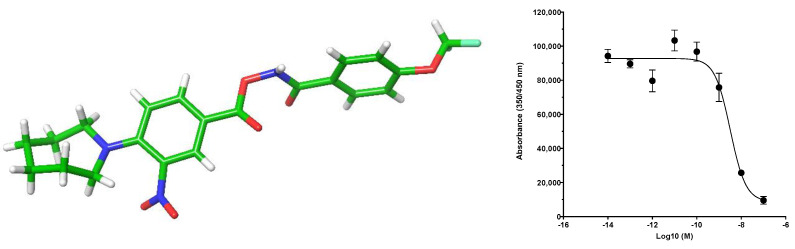
Single-crystal X-ray structure and dose–response curve of **1** in cell lysate assay.

To check whether biological activity was driven either by hydroxamic acid chelation of Zn^2+^ or due to the metal impurities present [13], **1** was re-tested in our cell lysate assay with the addition of the general metal chelator ethylenediaminetetraacetic acid (EDTA) and, separately, *N*,*N*,*N*′,*N*′-tetrakis(2-pyridinylmethyl)-1,2-ethanediamine, a high-affinity chelator of Zn^2+^. The IC_50_ of **1** remained consistent in both cases (7 nM and 8 nM, respectively), thus ruling out metal chelation/contamination. Further confirmation of the correct structure and lack of metal ions present came from a single-crystal X-ray structure solved following slow vapour diffusion crystallisation (Figure 3).

## 3. Mechanism of Action

Almost all reported small-molecule inhibitors of acid ceramidase are covalent in their mechanism of action by reaction with cysteine 143 [14,15,16,17,18,19,20,21,22]. In some cases, the covalent warhead itself is linked to promiscuity and intrinsic chemical instability. To probe whether **1** was chemically reactive in this way, a sample was stirred with the amino acid cysteine. LCMS analysis indicated that **1** slowly decomposed into two main chemical species; however, neither mass corresponded to the expected addition of cysteine (see Supplementary Material).

**Figure 4:**
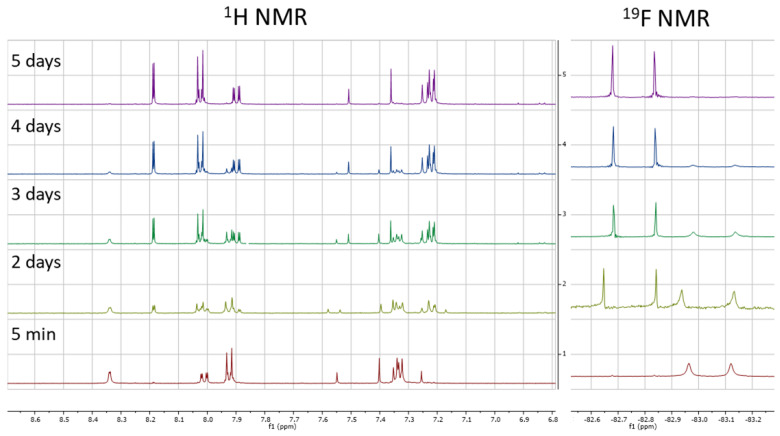
^1^H and ^19^F NMR studies monitoring the reaction of 1 with *d*6-DMSO.

This result prompted us to check the stability of the DMSO stock solution of **1**. Although clean once freshly prepared, it was clear that the sample slowly degraded into two species when left for extended periods of time at room temperature (see Supplementary Material). This partly degraded sample was again tested in our cell lysate assay and gave slightly reduced IC_50_ values relative to the freshly prepared sample. One of the degradation species was identified as the carboxylic acid **12**, exhibiting the same retention time and mass by LCMS in addition to exhibiting the same Rf by TLC analysis (see Supplementary Material). The identification of the other chemical species proved more difficult. Key to this was running LCMS analysis on a *d*6-DMSO NMR sample that was left at room temperature for 6 weeks. Despite also degrading into the carboxylic acid **12**, the mass of the other species was different by +6 Daltons compared to the non-deuterated sample. This not only strongly suggested that the other side of the molecule had reacted with *d*6-DMSO, but that both CD_3_ units were present. To monitor the speed of degradation, a fresh sample was analysed periodically by ^1^H and ^19^F NMR spectroscopy (Figure 4). The relative integrations of **1**, **12,** and **13** were plotted against time, which gave an estimated half-life of just over two days (see Supplementary Material).

**Scheme 3:**
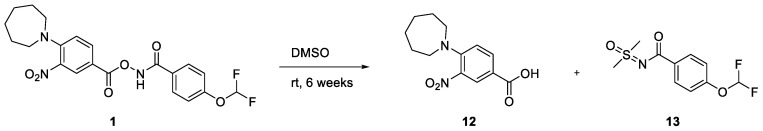
Degradation of **1** in DMSO.

**Figure 5:**
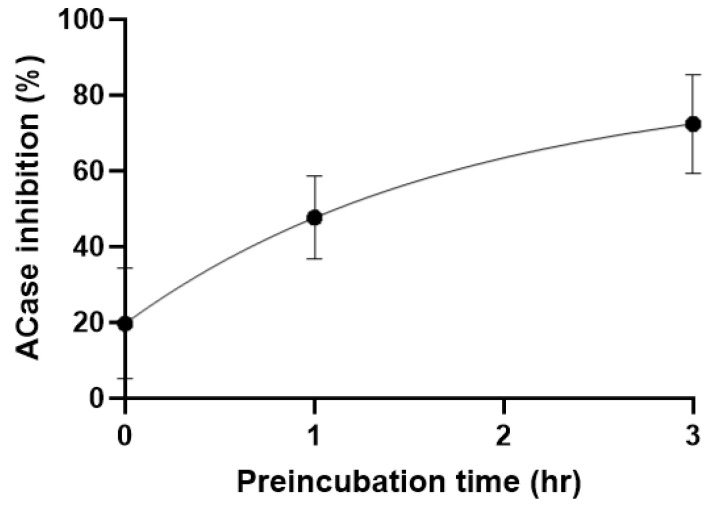
Preincubation experiment at IC_50_ concentration.

A partially degraded sample was purified by reverse-phase chromatography to isolate the DMSO-addition species. Although only a small amount of material was isolated, ^1^H NMR spectroscopic analysis was consistent with the proposed structure (Scheme 3). The ^1^H NMR data, in particular, were in line with that reported for a related compound (*3f*, see Supplementary Material) [23]. Such reactivity has been reported between *N*-pivaloyloxybenzamides and sulfoxides in the presence of an iron catalyst [23]. The decomposition reaction to form a sulfoximine reported in this paper should be of interest to the synthetic community as it proceeds cleanly at ambient temperature without the need for reagents nor a catalyst; however, further optimisation of the reaction is beyond the scope of this paper. **13** was tested in our cell lysate assay and showed no activity, suggesting that the original hit compound **1** was indeed the active species, likely reacting covalently with an active site cysteine amino acid residue of acid ceramidase. To confirm this, **1** was pre-incubated at 0, 1, and 3 h, showing the time-dependent inhibition pattern expected of a covalent inhibitor (Figure 5).

Recent in silico modelling efforts to find suitable replacements to the *N*-(benzoyloxy)benzamide scaffold have been reported, which could de-risk this motif [24], but our current decision is to not pursue **1** further.

## 4. Conclusions

Following HTS efforts, **1** was identified as an extremely potent inhibitor of acid ceramidase. Despite this, following resynthesis and stability studies of **1**, it became apparent that the active compound was not chemically stable and its degradation products were biologically inactive. **1** reacts with DMSO at room temperature over time and is very likely an extremely promiscuous compound which is unsuitable as a starting point in drug discovery. The reported biological activity of *N*-(benzoyloxy)benzamides and their analogues should be treated with caution and we suggest removal of such compounds from screening libraries in future.

## Data Availability

The data presented in this study are openly available in [CSD], reference number [CCDC 2378373].

## Supplementary Materials

The following supporting information can be downloaded at https://www.mdpi.com/article/10.3390/molecules29215143/s1. All chemical and biological experimental procedures can be found in the supplementary information.
