# Supplementary material for: Beware of N-Benzoyloxybenzamides
Source: Molecules. 2024 Oct 31;29(21):5143. doi: 10.3390/molecules29215143 (PMC11548001; doi:10.3390/molecules29215143)
Supplement: Supplementary file 1 [file molecules-29-05143-s001.zip › molecules-3214020-supplementary.pdf]

*Electronic Supplementary Information*

# Beware of *N*-benzoyloxybenzamides

Jonathan Cubitt, Mari Davies, Ross Riseley, Gabrielle Evans, Sian E. Gardiner, Benson M. Kariuki†, Simon E. Ward, Emyr Lloyd-Evans, Helen Waller-Evans* and D. Heulyn Jones*,†

**Medicines Discovery Institute, Cardiff University, Main Building, Park Place, Cardiff, CF10 3AT, U.K.*
*†Chemistry Department, Cardiff University, Cardiff CF10 3AT, UK*

# Contents

**Experimental procedures** 3-5

**Copies of $^{1}H$, $^{13}C$ and $^{19}F$ NMR and UPLC spectra**

4-(difluoromethoxy)benzenecarbohydroxamic acid **10** 6

4-(azepan-1-yl)-3-nitro-benzoic acid **12** 8

[[4-(difluoromethoxy)benzoyl]amino]4-(azepan-1-yl)-3-nitro-benzoate **1** 10

4-(difluoromethoxy)-N-[dimethyl(oxo)-λ6-sulfanylidene]benzamide **13** 12

1,3-bis(4-(difluoromethoxy)phenyl)urea **8** 14

Copies of UPLC spectra Stability Studies + TLC 16-18

NMR stability study 19

Biological assays 20

**Experimental Procedures**

4-(Difluoromethoxy*)*benzenecarbohydroxamic acid **1**

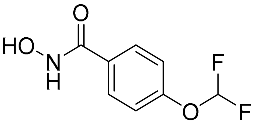

**Step 1** - A mixture of thionyl chloride (2.0 mL, 27.42 mmol) and 4-(difluoromethoxy)benzoic acid (250 mg, 1.33 mmol) was heated to 65°C for 3 hours. The solvent was removed under reduced pressure in a fume cupboard, providing the 4(difluoromethoxy)benzoyl chloride without further purification.

**Step 2** - A mixture hydroxylamine hydrochloride (185 mg, 2.66 mmol), potassium carbonate (373 mg, 2.66 mmol), ethyl acetate (5 mL), and water (5 mL) were added in sequence listed. After cooling to 0°C, a solution of 4-(difluoromethoxy)benzoyl chloride (274.5 mg) in ethyl acetate (5 mL) was added dropwise. After stirring at room temperature for 16 h, layers were separated, and the aqueous layer was extracted with ethyl acetate (2 × 15 ml). The combined organic layer was successively washed with water (2 × 15 ml), brine (2 × 10 ml), and dried over anhydrous $MgSO_4$, filtered and the solvent removed under reduced pressure, to give the crude product which was purified by automated column chromatography on silica (ISCO, 24 g, gradient from petroleum ether to ethyl acetate over 30 CV) to give the titled compound **XX** (237 mg, 0.887 mmol, 86% yield) a colourless solid. $^1$H NMR (500 MHz, DMSO-*d6*) δ 11.23 (s, 1H), 9.05 (s, 1H), 7.85 – 7.78 (m, 2H), 7.33 (t, $J$ = 73.7 Hz, 1H), 7.26 – 7.21 (m, 2H). $^{19}$F NMR (471 MHz, DMSO-*d6*) δ -82.73 (d, $J$ = 73.8 Hz). $^{13}$C NMR (126 MHz, DMSO-*d6*) δ 163.2, 153.0, 129.4, 129.0, 118.1, 116.1 (t, $J$ = 258.3 Hz). ACQUITY UPLC® BEH C18 1.7 µm: Rt = 1.60 min; m/z 204.1, $C_8H_7F_2NO_3$ requires 204.1, found 204.1 $[M+H]^+$

4-(Azepan-1-yl)-3-nitro-benzoic acid **12**

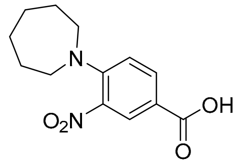

A mixture of 4-chloro-3-nitrobenzoic acid (500 mg, 2.48 mmol), hexamethyleneimine (0.30 mL, 2.48 mmol) and *N*-ethyldiisopropylethylamine (1.0 mL, 6.2 mmol) was heated in a Biotage microwave at 110°C in acetonitrile (8.0 mL) for 1 h. UPLC analysis showed some conversion to the desired product. Additional hexamethyleneimine (0.30 mL, 2.48 mmol) was added and the mixture heated for a further 2 h. Finally, additional hexamethyleneimine (0.30 mL, 2.48 mmol) was added once more, and the reaction heated for a final 3 h with UPLC analysis confirming reaction completion. The mixture was concentrated under reduced pressure and the residue redissolved in EtOAc (100 mL) and washed with water (3 × 50 mL), 1M HCl (aq) (3 × 50 mL). The organic phase was dried over $MgSO_4$, filtered and concentrated under reduced pressure to give the product, **12**, without the need for further purification (526 mg, 80% yield) as a yellow solid. $^1$H NMR (500 MHz, DMSO-*d6*) δ 12.81 (s, 1H), 8.18 (d, $J$ = 2.2 Hz, 1H), 7.90 (dd, $J$ = 9.1, 2.2 Hz, 1H), 7.24 (d, $J$ = 9.1 Hz, 1H), 3.30 – 3.26 (m, 4H), 1.80 – 1.71 (m, 4H), 1.52 – 1.44 (m, 4H). $^{13}$C NMR (126 MHz, , DMSO-*d6*) δ 165.7 (quat C) 147.1 (quat C) 136.0 (quat C), 133.0 (CH), 128.3 (CH), 117.4 (CH), 50.9 (2 × $CH_2$), 27.0 (2 × $CH_2$), 26.9 (2 × $CH_2$) missing a quat C due to weak sample.

ACQUITY UPLC® BEH C18 1.7 µm: Rt = 1.71 min; m/z 265.1; HRMS(EI) $C_{13}H_{17}N_2O_4$ requires 265.1193, found 265.1188 $[M+H]^+$.

[[4-(difluoromethoxy)benzoyl]amino]4-(azepan-1-yl)-3-nitro-benzoate **1**

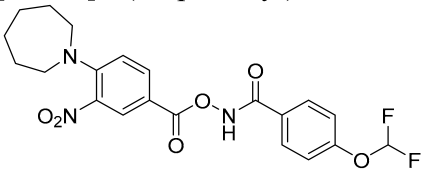

**1**

Step 1 - A mixture of Thionyl chloride (2.0 mL, 27.4 mmol) and 4-(azepan-1-yl)-3-nitro-benzoic acid (124 mg, 0.47 mmol) was heated to 65°C for 3 hours. The solvent was removed under reduced pressure in a fume cupboard, providing 4-(azepan-1-yl)-3nitro-benzoyl chloride which was used without further purification.

Step 2 – To a solution of 4-(difluoromethoxy)benzenecarbohydroxamic acid (100 mg, 0.49 mmol), diisopropylethylamine (0.10 mL, 0.74 mmol) in DCM (2.0 mL), was added a solution of 4-(azepan-1yl)-3-nitro-benzoyl acid (98 mg, 0.37 mmol) in DCM (2.0 mL) dropwise. The reaction mixture was diluted with DCM (30 mL) and washed with water (2 × 15 mL), 1M HCl (aq) (3 × 20 mL). the organic phase was dried over $MgSO_4$, filtered and concentrated under reduced pressure to give the crude product, which was purified by automated column chromatography on silica (ISCO, 24 g, gradient from petroleum ether to ethyl acetate over 35 CV) and then by automated reverse-phase column chromatography on silica (AquiPrep, 30 min run from water to methanol) to give titled compound **1** (40 mg, 0.084 mmol, 18% yield) as a yellow solid. $^1$H NMR (500 MHz, DMSO-*d6*) δ 12.61 (s, 1H), 8.34 (d, *J* = 2.2 Hz, 1H), 8.01 (dd, *J* = 9.2, 2.3 Hz, 1H), 7.95 – 7.91 (m, 2H), 7.39 (t, *J* = 73 Hz, 1H) 7.36 – 7.32 (m, 3H), 3.36 – 3.32 (m, 4H), 1.80 (m, 4H), 1.50 (app p, *J* = 2.6 Hz, 4H). $^{13}$C NMR (126 MHz, DMSO-*d6*) δ 163.9 (quat C), 163.0 (quat C), 153.9 (quat C), 148.0 (quat C), 136.2 (quat C), 133.0 (CH), 129.6 (CH), 129.1 (CH), 127.5 (quat C), 118.0 (CH), 116.0 (t, *J* = 425.9 Hz, CH), 114.0 (CH), , 51.1 (2 × $CH_2$), 27.1 (2 × $CH_2$), 27.0 (2 × $CH_2$), missing quat C; $^{19}$F NMR (471 MHz, DMSO-d6) δ -83.02 (d, *J* = 73.6 Hz); $C_{21}H_{21}F_2N_3O_6$ requires 450.1, ACQUITY UPLC® BEH C18 1.7 µm: Rt = 1.83 min; m/z 450.2, $[M+H]^+$ was found.

4-(Difluoromethoxy)-*N*-[dimethyl(oxo)-λ6-sulfanylidene]benzamide **13**

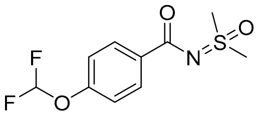

**13**

[[4-(difluoromethoxy)benzoyl]amino]4-(azepan-1-yl)-3-nitro-benzoate (10.0 mg, 0.02 mmol) was left in DMSO for 7 weeks until it degraded fully into titled compound **13** and 4-(azepan-1-yl)-3-nitrobenzoic acid. The solution was purified directly by reverse phase automatic column chromatography on silica (AquiPrep, 1 h gradient from water to MeOH) to give titled compound **13** (2.5 mg, 0.009 mmol, 42% yield assuming full degradation) as a yellow solid. $^1$H NMR (500 MHz Chloroform-*d3*) δ 8.19 – 8.08 (m, 2H), 7.14 – 7.08 (m, 2H), 6.57 (t, *J* = 73.5 Hz, 1H), 3.39 (s, 6H); $^{19}$F NMR (471 MHz, Chloroform-*d3*) δ -81.37 (d, *J* = 73.1 Hz);

$C_{10}H_{11}F_2NO_3S$ requires 264.1, ACQUITY UPLC® BEH C18 1.7 µm: Rt = 1.48 min; m/z 264.0 $[M+H]^+$ was found.

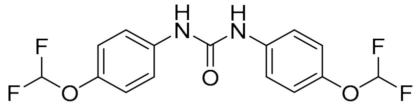

1,3-bis(4-(difluoromethoxy)phenyl)urea **8**

1-(Difluoromethoxy)-4-isocyanato-benzene (50 mg, 0.27 mmol) was pipetted into water (2.5 mL) and the mixture was stirred overnight at room temperature. A white precipitate had formed, which was collected by gravity filtration. The solid collected was dried in a vacuum oven set at 40 °C to give **8** [4-(Difluoromethoxy)phenyl]carbamic acid (50 mg, 0.234 mmol, 87% yield) as a colourless solid; $^1$H NMR (500 MHz, DMSO-*d*6) δ 8.73 (s, 1H), 7.49 – 7.41 (m, 2H), 7.13 – 7.09 (m, H), 7.09 (t, $J$ = 74.6 Hz, 1H); $^{13}$C NMR (126 MHz, DMSO-*d6*) δ 152.8 (C), 145.6-145.5 (C, multiplet), 137.1 (C, t, $J$ = 8.8 Hz), 119.7 (CH), 119.6 (CH), 116.5 (CH, t, $J$ = 258.3 Hz) $^{19}$F NMR (471 MHz, DMSO-*d*6) δ -81.37 (d, $J$ = 73.1 Hz); ACQUITY UPLC® BEH C18 1.7 µm: Rt = 1.67 min; m/z 345.1 $[M+H]^+$.

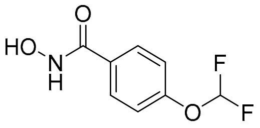
HO
N
H
O
O
F
F

**10**

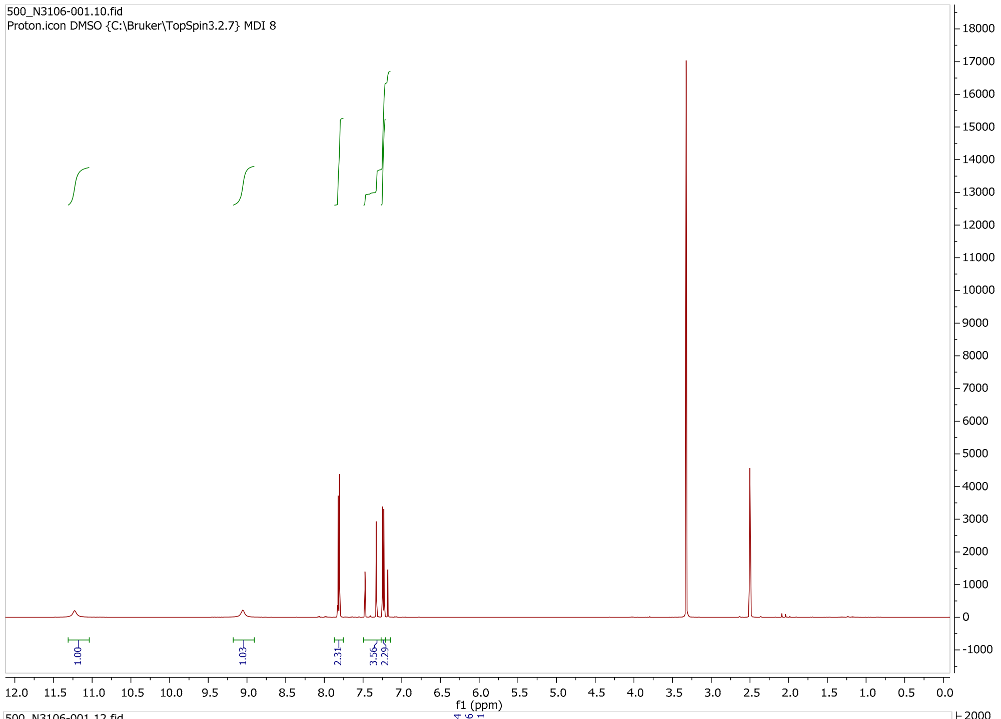
500_N3106-001.10.fid
Proton.icon DMSO {C:\Bruker\TopSpin3.2.7} MDI 8
f1 (ppm)

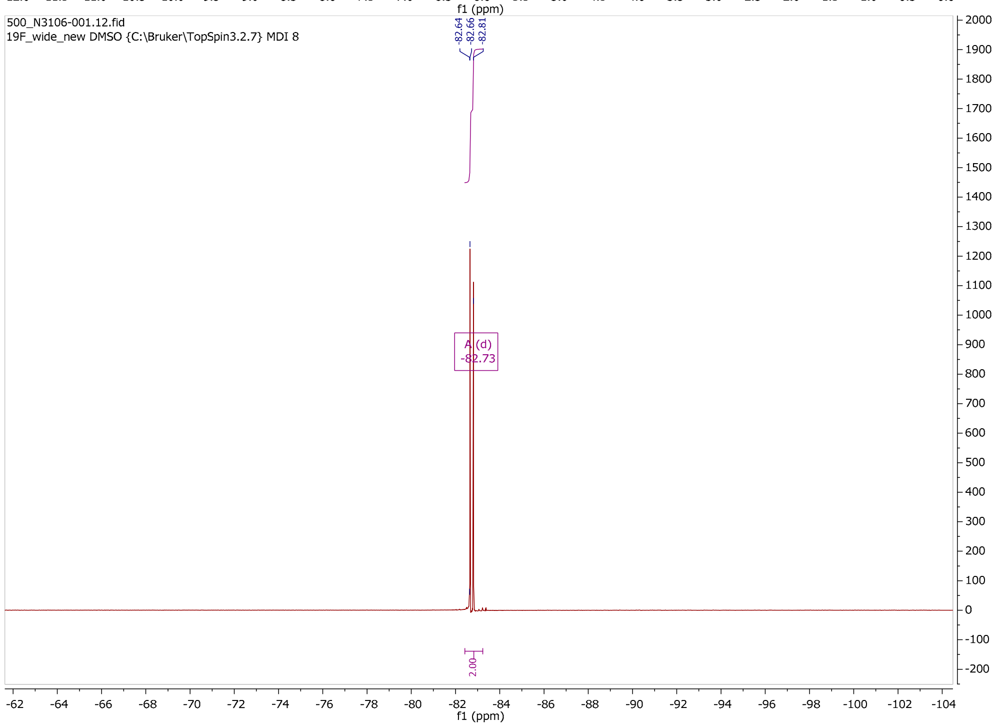
500_N3106-001.12.fid
19F_wide_new DMSO {C:\Bruker\TopSpin3.2.7} MDI 8
A (d)
-82.73
f1 (ppm)

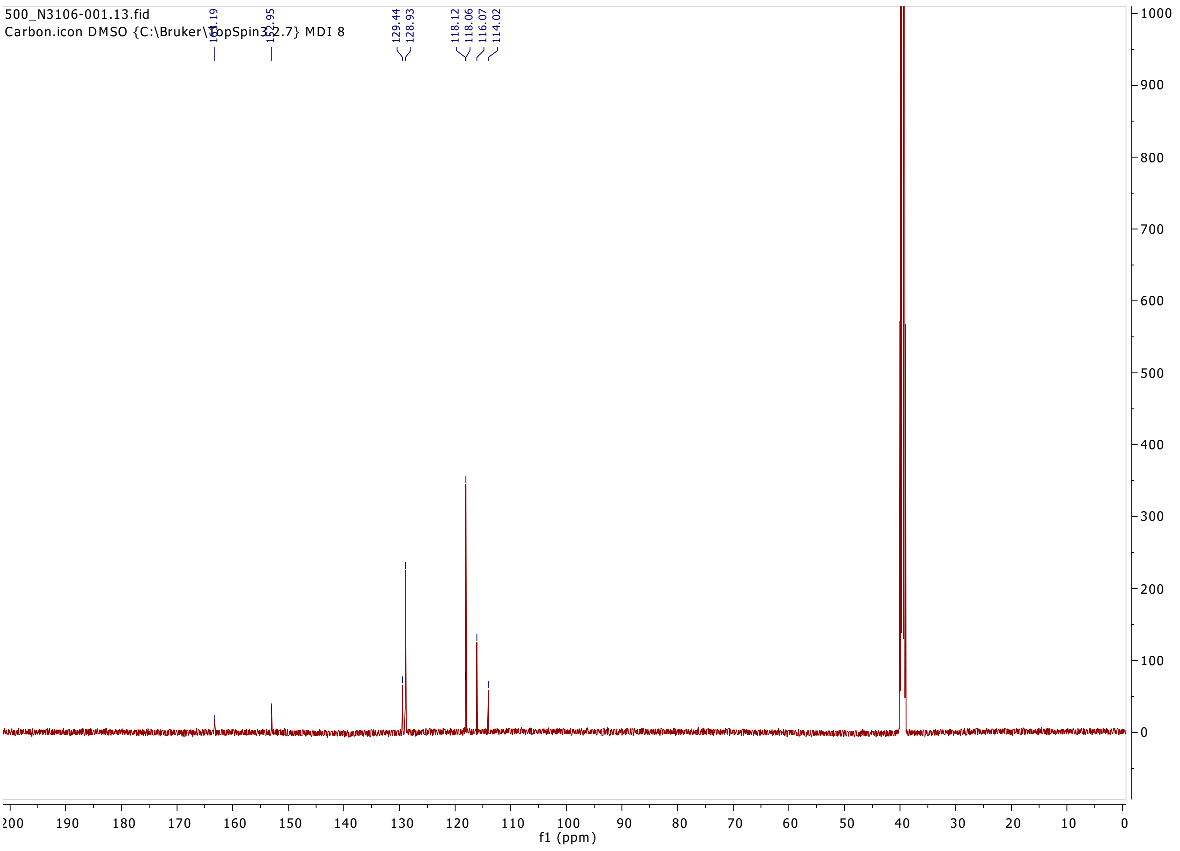
500_N3106-001.13.fid
Carbon.icon DMSO {C:\Bruker\TopSpin3.2.7} MDI 8
129.44
128.93
118.12
118.06
116.07
114.02
f1 (ppm)

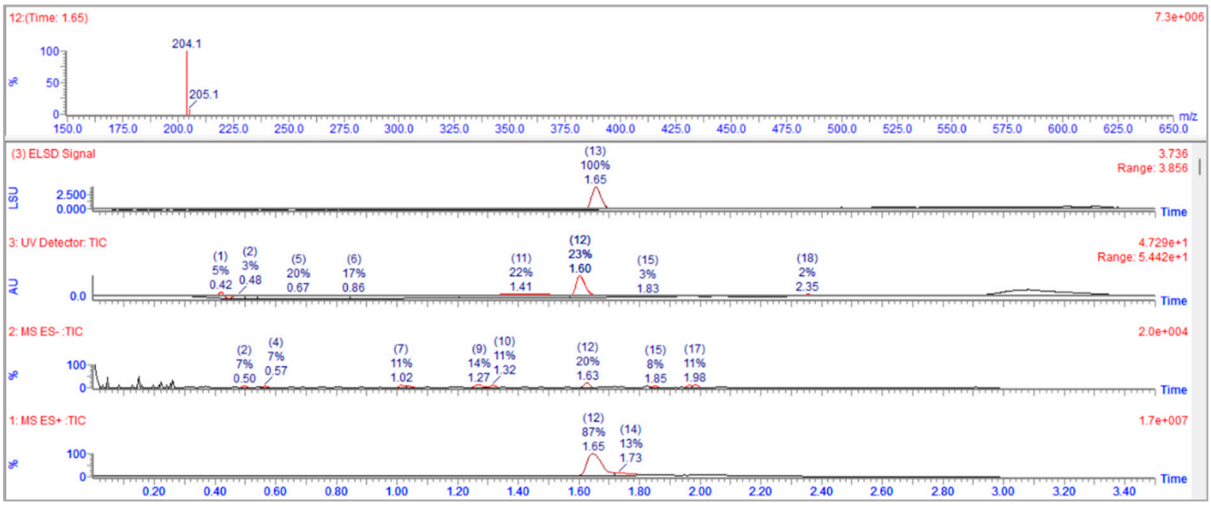
12:(Time: 1.65)
7.3e+006
204.1
205.1
m/z
(3) ELSD Signal
3.736
Range: 3.856
(13)
100%
1.65
3: UV Detector: TIC
4.729e+1
Range: 5.442e+1
2: MS ES- :TIC
2.0e+004
1: MS ES+ :TIC
1.7e+007
Time

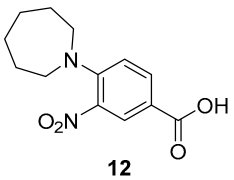
N
O2N
OH
O

**12**

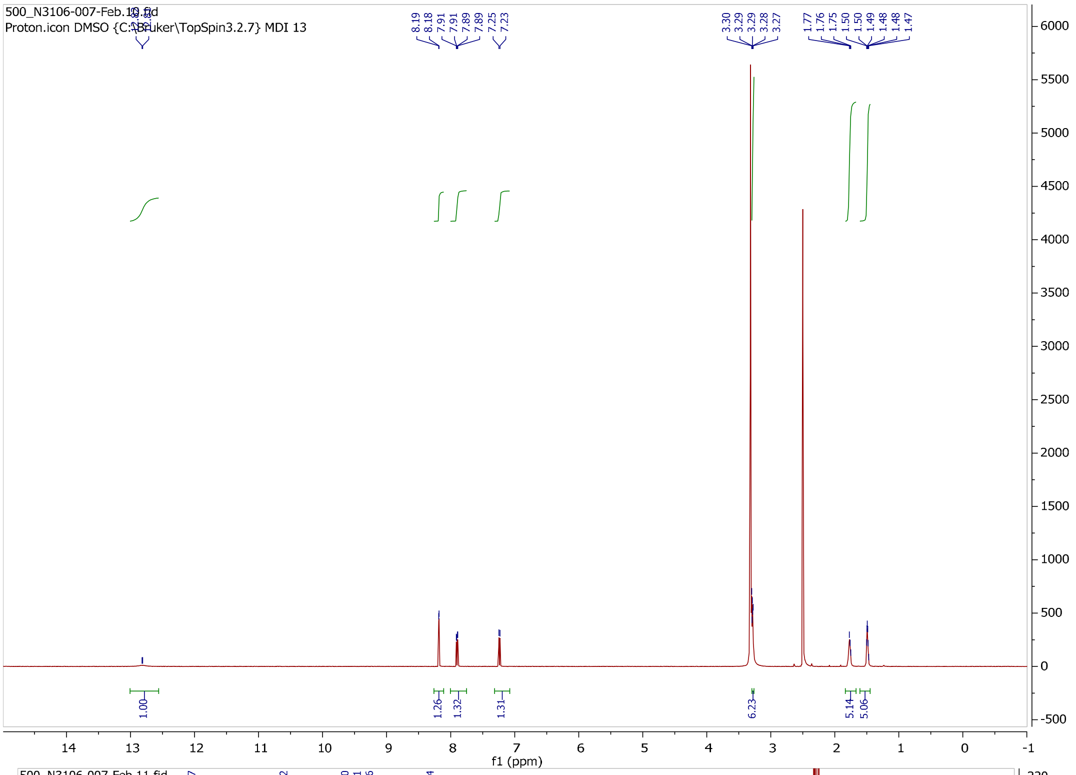
500_N3106-007-Feb.10.fid
Proton.icon DMSO {C:\Bruker\TopSpin3.2.7} MDI 13
8.19
8.18
7.91
7.91
7.89
7.89
7.25
7.23
3.30
3.29
3.29
3.28
3.27
1.77
1.76
1.75
1.50
1.50
1.49
1.48
1.48
1.47
1.00
1.26
1.32
1.31
6.23
5.14
5.06
f1 (ppm)

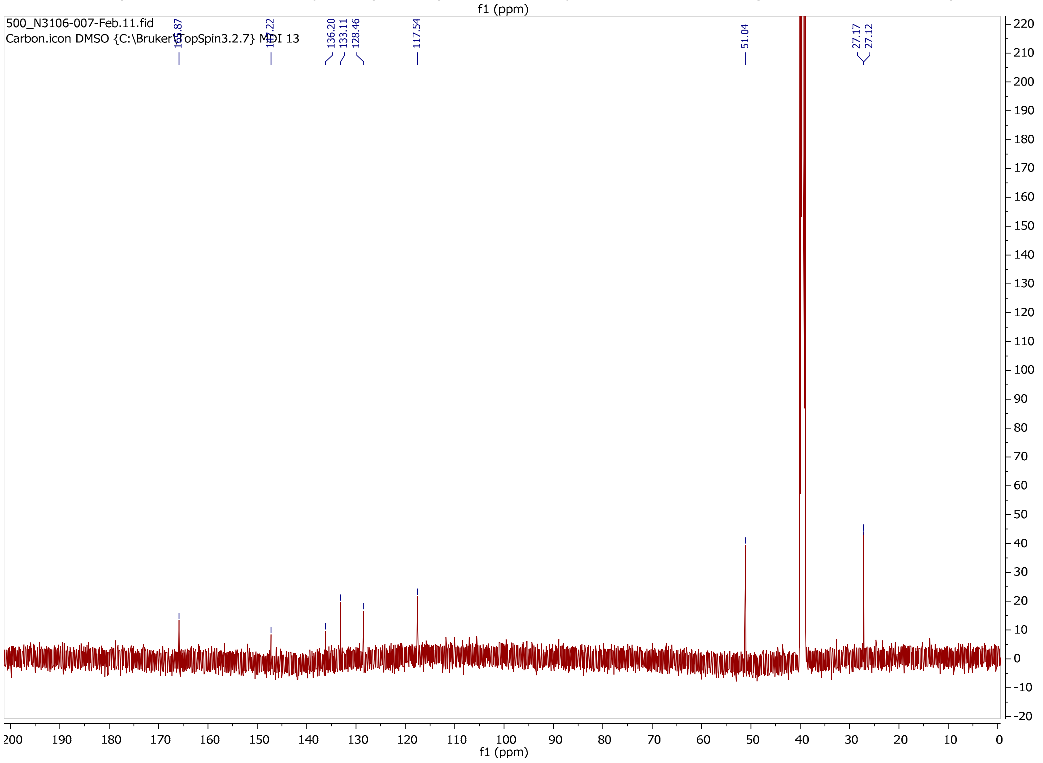
500_N3106-007-Feb.11.fid
Carbon.icon DMSO {C:\Bruker\TopSpin3.2.7} MDI 13
136.20
133.11
128.46
117.54
51.04
27.17
27.12
f1 (ppm)

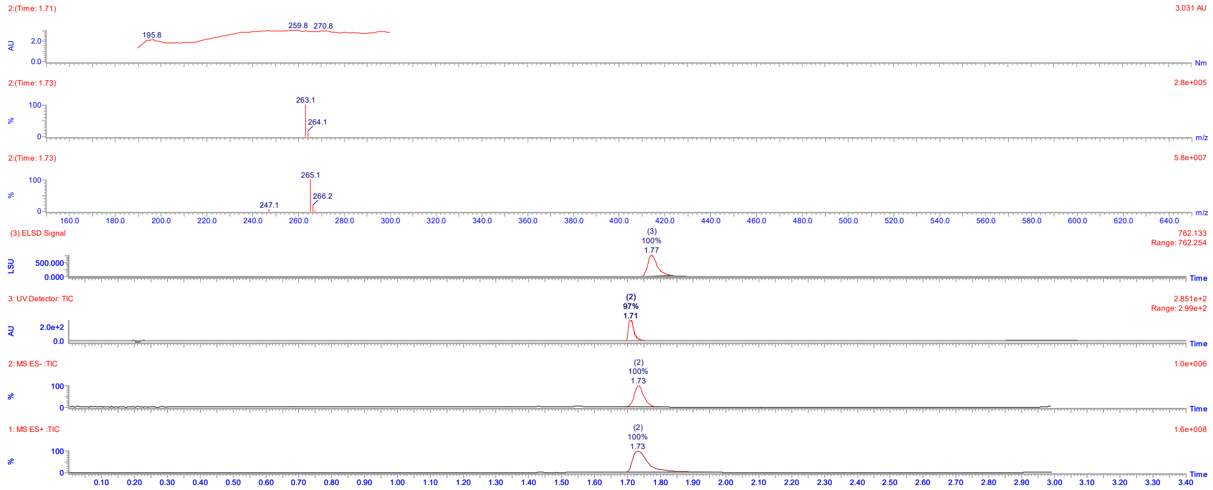


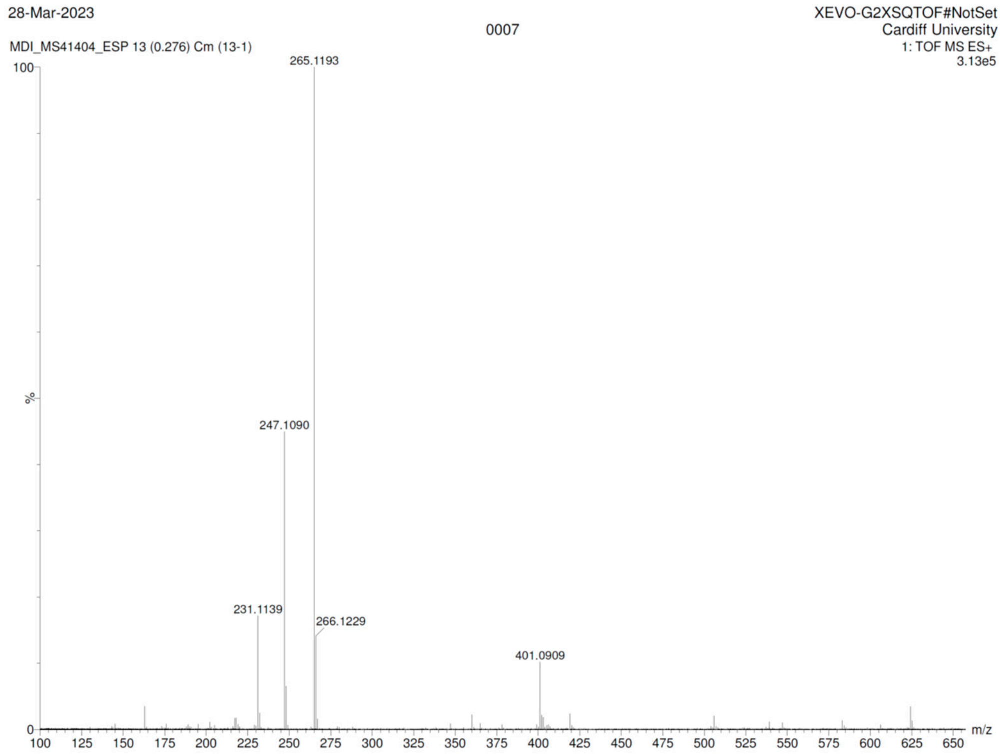


**Elemental Composition Report** **Page 1**

**Single Mass Analysis**
Tolerance = 10.0 PPM / DBE: min = -1.5, max = 100.0
Element prediction: Off
Number of isotope peaks used for i-FIT = 4

Monoisotopic Mass, Odd and Even Electron Ions
13 formula(e) evaluated with 1 results within limits (all results (up to 1000) for each mass)
Elements Used:
C: 0-13 H: 0-17 N: 0-2 O: 0-4

| | | | | | | | | |
|---|---|---|---|---|---|---|---|---|
| Minimum: | | | | -1.5 | | | | |
| Maximum: | | 5.0 | 10.0 | 100.0 | | | | |
| Mass | Calc. Mass | mDa | PPM | DBE | i-FIT | Norm | Conf(%) | Formula |
| 265.1193 | 265.1188 | 0.5 | 1.9 | 6.5 | 448.4 | n/a | n/a | C13 H17 N2 O4 |

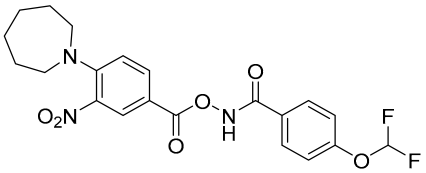
N
O₂N
O
O
N
H
O
F
F
O

**1**

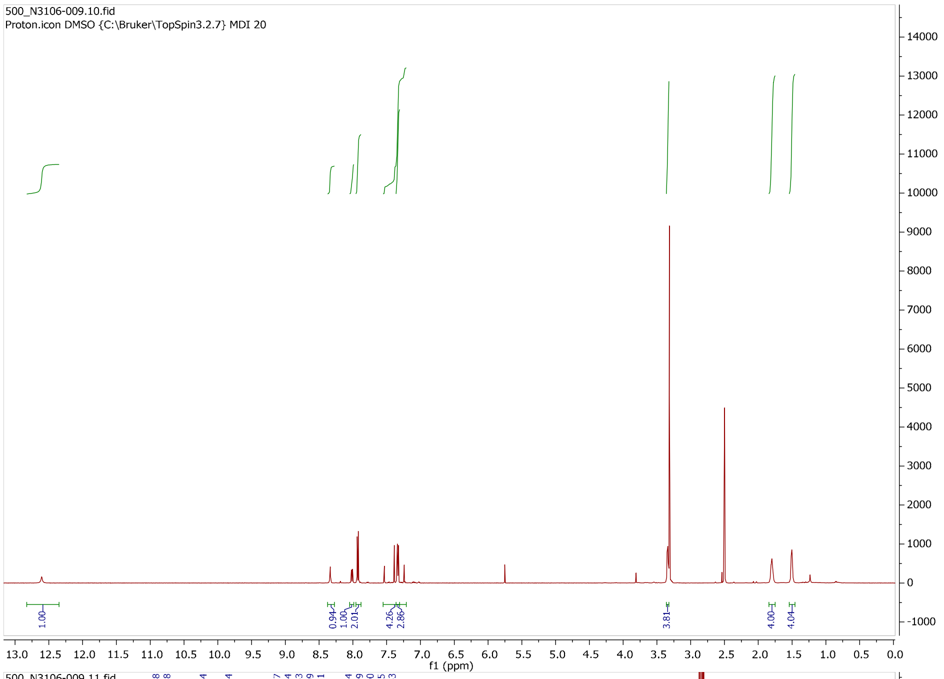
500_N3106-009.10.fid
Proton.icon DMSO {C:\Bruker\TopSpin3.2.7} MDI 20
1.00
0.94
1.00
2.01
4.26
2.86
3.81
4.00
4.04
f1 (ppm)

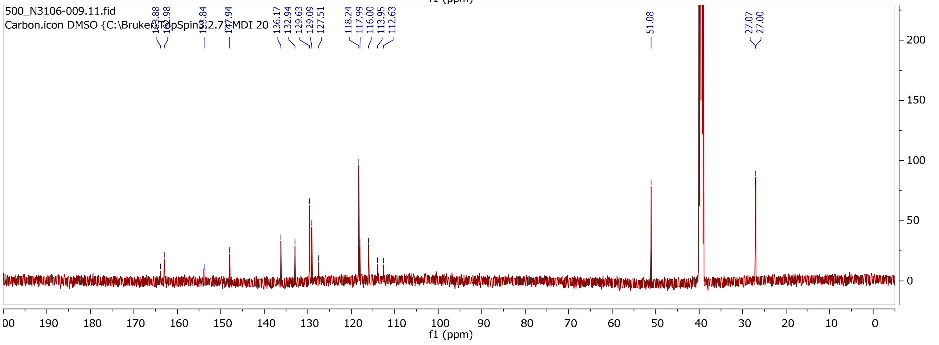
500_N3106-009.11.fid
Carbon.icon DMSO {C:\Bruker\TopSpin3.2.7} MDI 20
162.98
136.17
132.94
129.63
129.09
127.51
118.24
117.99
116.00
113.95
112.63
51.08
27.07
27.00
f1 (ppm)

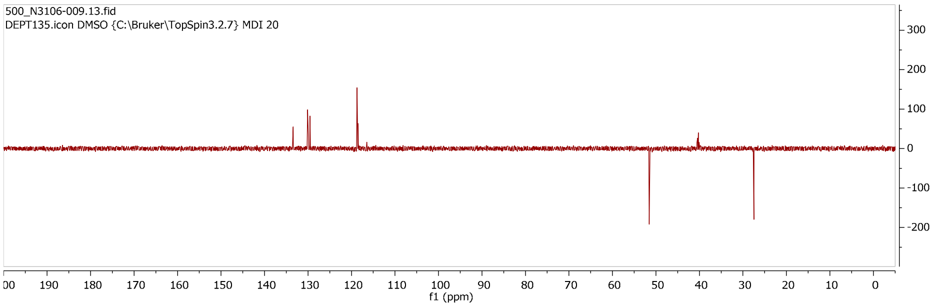
500_N3106-009.13.fid
DEPT135.icon DMSO {C:\Bruker\TopSpin3.2.7} MDI 20
f1 (ppm)

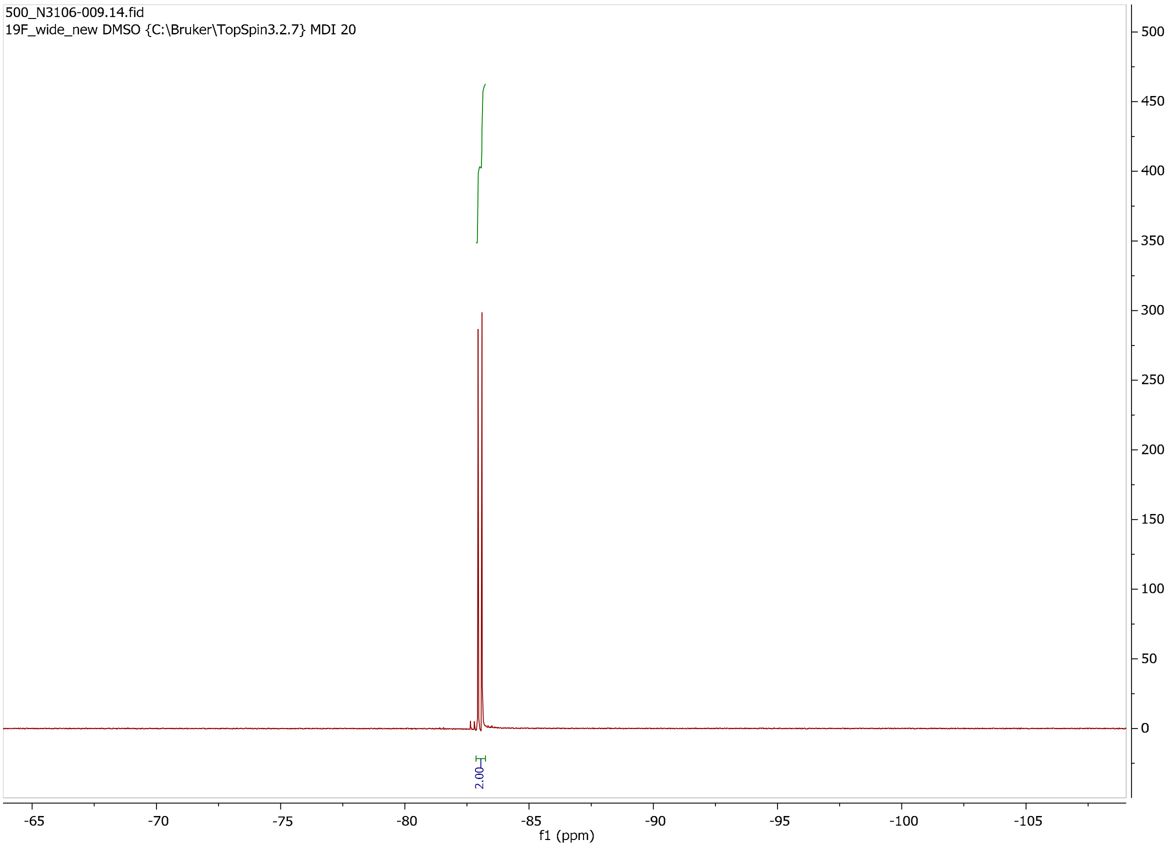
500_N3106-009.14.fid
19F_wide_new DMSO {C:\Bruker\TopSpin3.2.7} MDI 20
2.00
f1 (ppm)

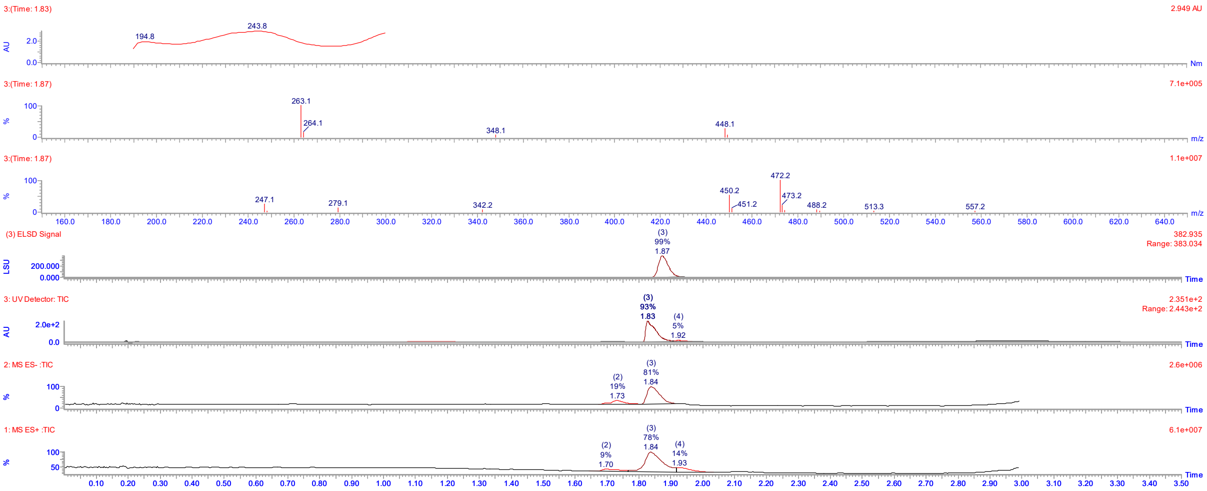
3:(Time: 1.83)
194.8
243.8
2.949 AU
3:(Time: 1.87)
263.1
264.1
348.1
448.1
7.1e+005
3:(Time: 1.87)
247.1
279.1
342.2
450.2
451.2
472.2
473.2
488.2
513.3
557.2
1.1e+007
(3) ELSD Signal
99%
1.87
382.935
Range: 383.034
3: UV Detector: TIC
93%
1.83
5%
1.92
2.351e+2
Range: 2.443e+2
2: MS ES- :TIC
19%
1.73
81%
1.84
2.6e+006
1: MS ES+ :TIC
9%
1.70
78%
1.84
14%
1.93
6.1e+007

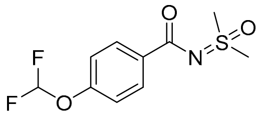
F
F
O
O
N
S
O

**13**

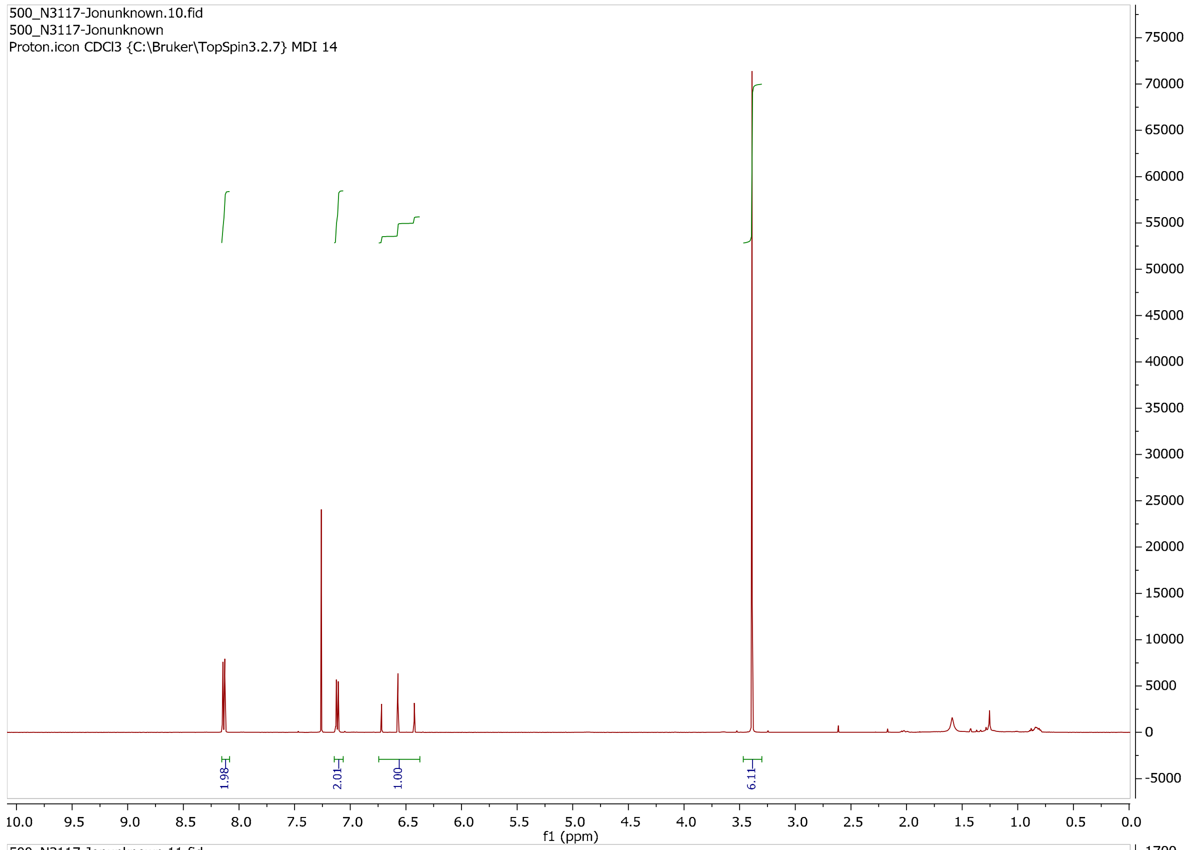
500_N3117-Jonunknown.10.fid
500_N3117-Jonunknown
Proton.icon CDCl3 {C:\Bruker\TopSpin3.2.7} MDI 14
1.98
2.01
1.00
6.11
f1 (ppm)

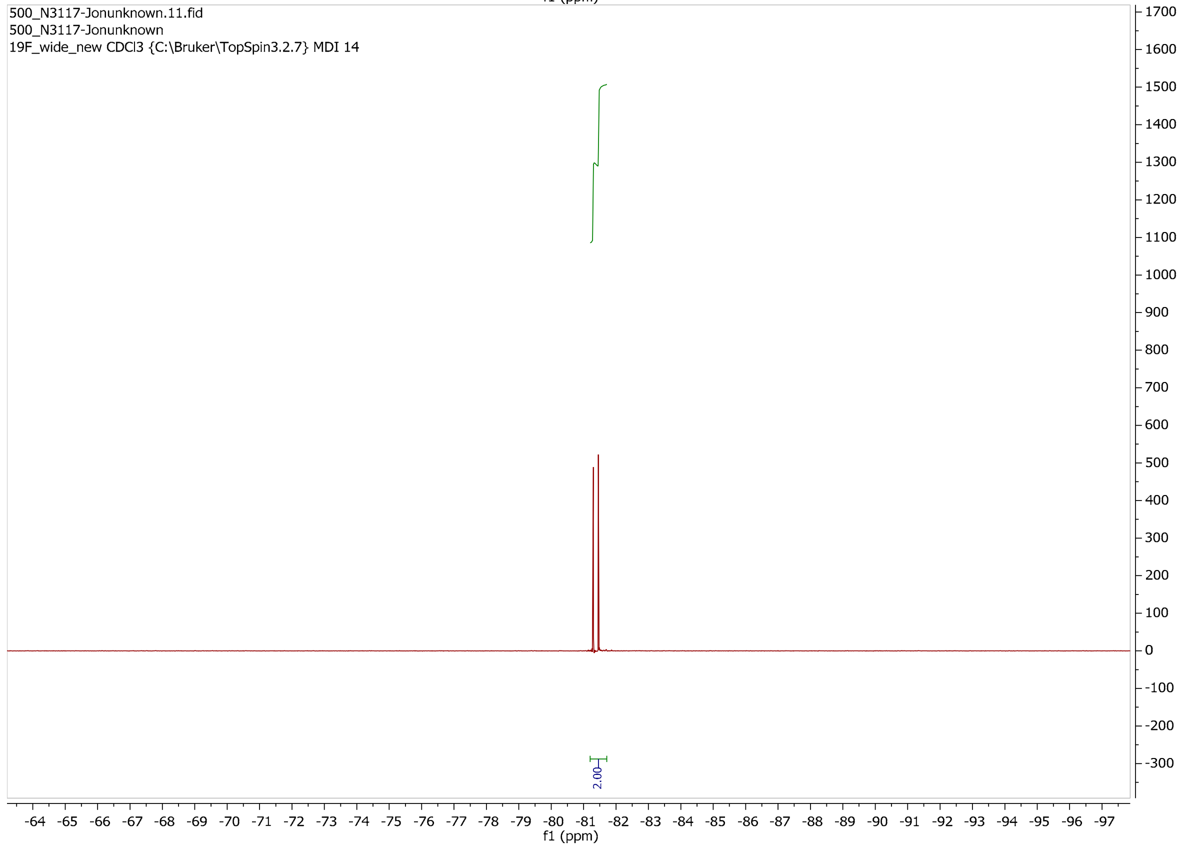
500_N3117-Jonunknown.11.fid
500_N3117-Jonunknown
19F_wide_new CDCl3 {C:\Bruker\TopSpin3.2.7} MDI 14
2.00
f1 (ppm)

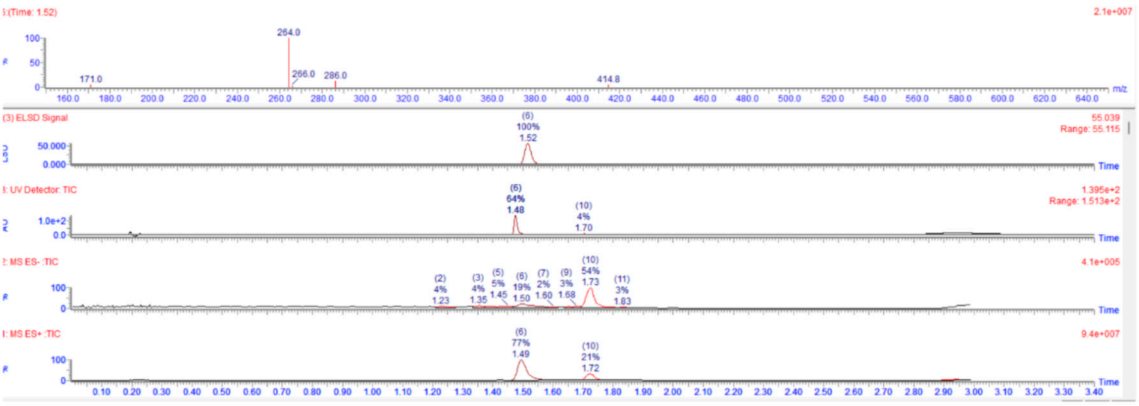

(Time: 1.52)
2.1e+007
264.0
171.0
266.0
286.0
414.8
m/z
(3) ELSD Signal
(6) 100% 1.52
55.039
Range: 55.115
UV Detector: TIC
(6) 64% 1.48
(10) 4% 1.70
1.395e+2
Range: 1.513e+2
MS ES- :TIC
(2) 4% 1.23
(3) 4% 1.35
(5) 5% 1.45
(6) 19% 1.50
(7) 2% 1.60
(9) 3% 1.68
(10) 54% 1.73
(11) 3% 1.83
4.1e+005
MS ES+ :TIC
(6) 77% 1.49
(10) 21% 1.72
9.4e+007
Time

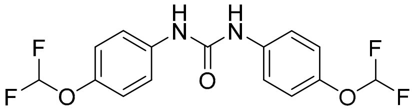

**8**

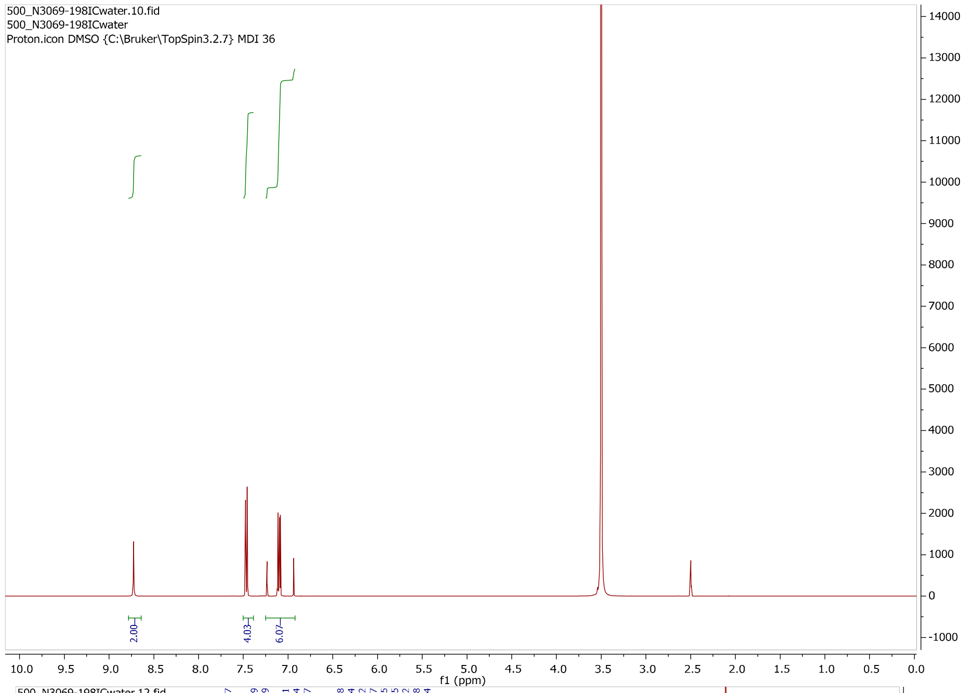
500_N3069-198ICwater.10.fid
500_N3069-198ICwater
Proton.icon DMSO {C:\Bruker\TopSpin3.2.7} MDI 36
2.00
4.03
6.07
f1 (ppm)

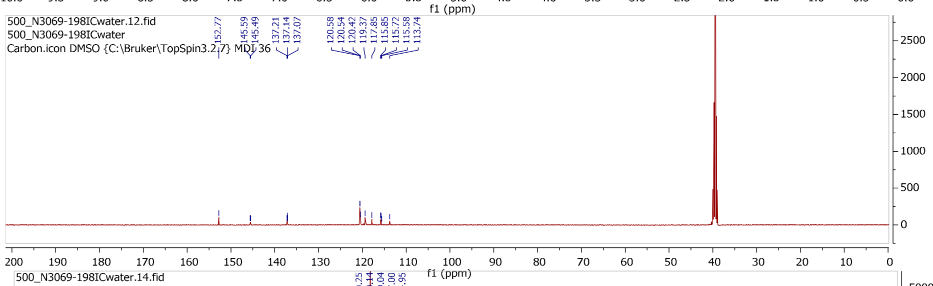
500_N3069-198ICwater.12.fid
500_N3069-198ICwater
Carbon.icon DMSO {C:\Bruker\TopSpin3.2.7} MDI 36
152.77
145.59
145.49
137.21
137.14
137.07
120.58
120.54
120.42
119.37
117.85
115.85
115.72
115.58
113.74
f1 (ppm)

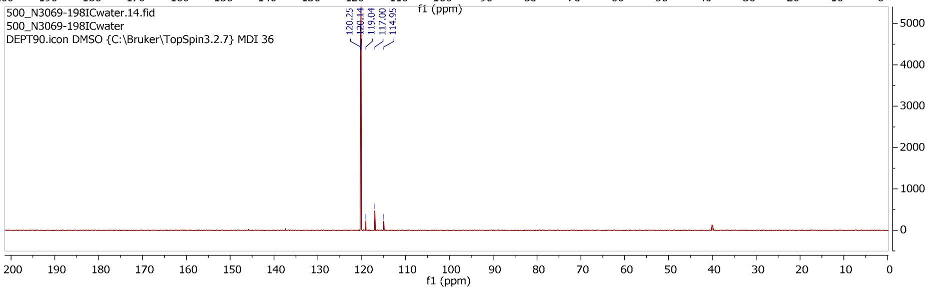
500_N3069-198ICwater.14.fid
500_N3069-198ICwater
DEPT90.icon DMSO {C:\Bruker\TopSpin3.2.7} MDI 36
120.25
119.04
117.00
114.95
f1 (ppm)

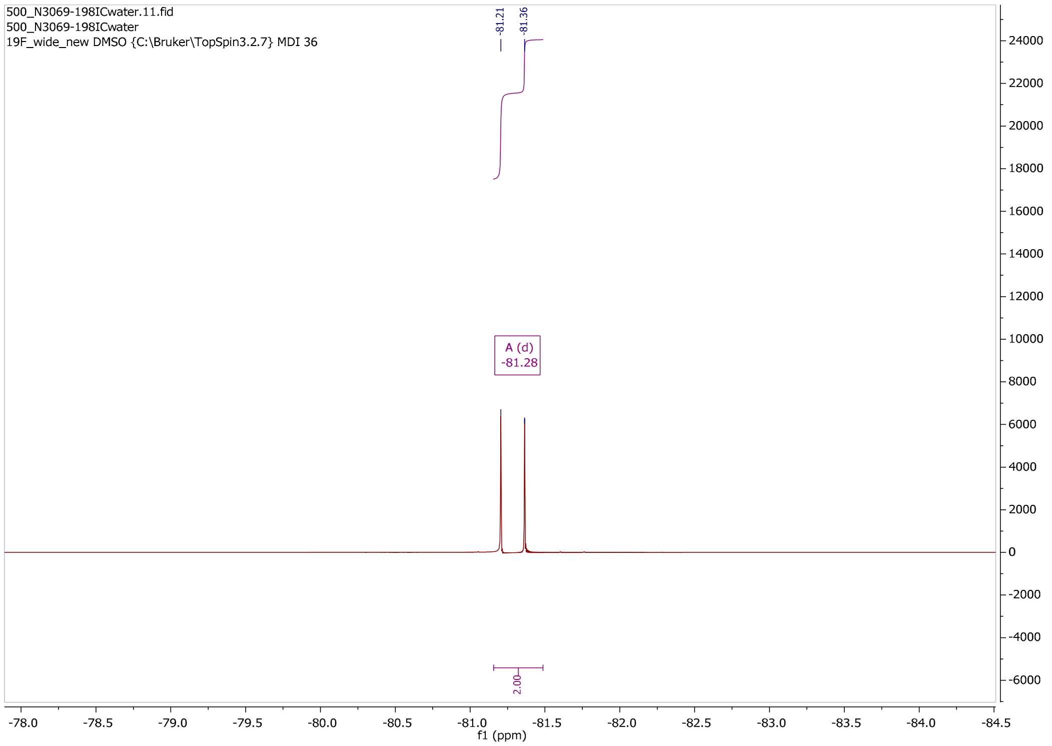
500_N3069-198ICwater.11.fid
500_N3069-198ICwater
19F_wide_new DMSO {C:\Bruker\TopSpin3.2.7} MDI 36
-81.21
-81.36
A (d)
-81.28
2.00
f1 (ppm)

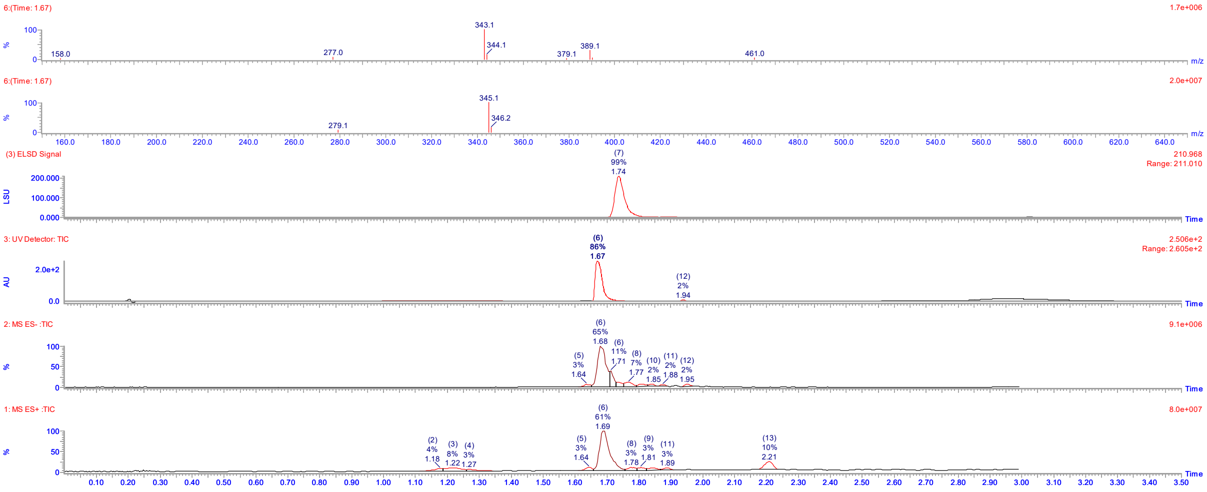
6:(Time: 1.67)
343.1
344.1
389.1
158.0
277.0
379.1
461.0
1.7e+006
6:(Time: 1.67)
345.1
346.2
279.1
2.0e+007
(3) ELSD Signal
(7)
99%
1.74
210.968
Range: 211.010
LSU
3: UV Detector: TIC
(6)
86%
1.67
(12)
2%
1.94
2.506e+2
Range: 2.605e+2
AU
2: MS ES- :TIC
9.1e+006
1: MS ES+ :TIC
8.0e+007
m/z
Time

# Degradation process (UPLC) Sample in DMSO stock at room temperature in FR code plastic tube

## 1 1 Hour after synthesis

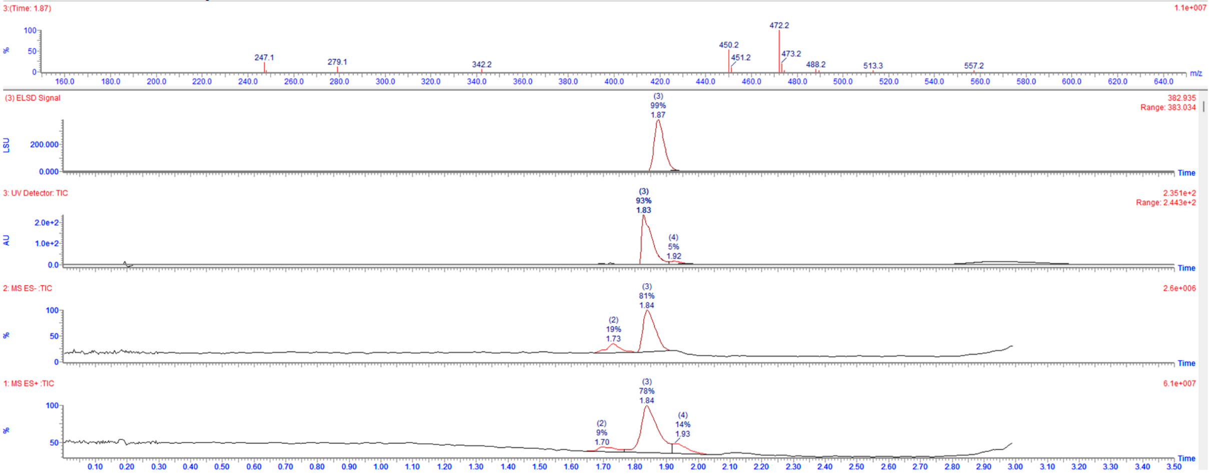


## 1 1 Week after synthesis

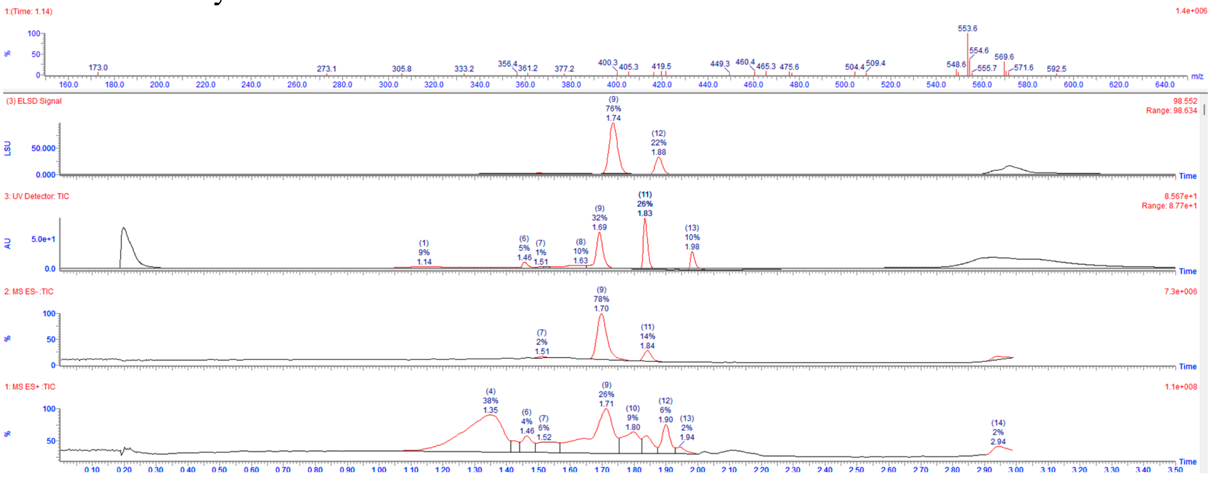


## 1 4 weeks after synthesis

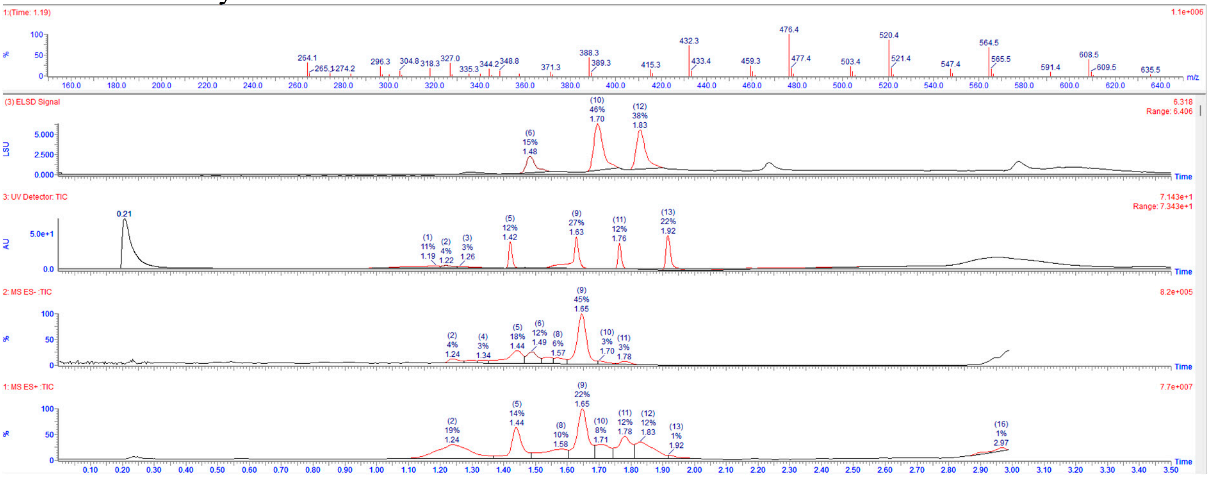

**1** in *d*6-DMSO solution left for 6 weeks

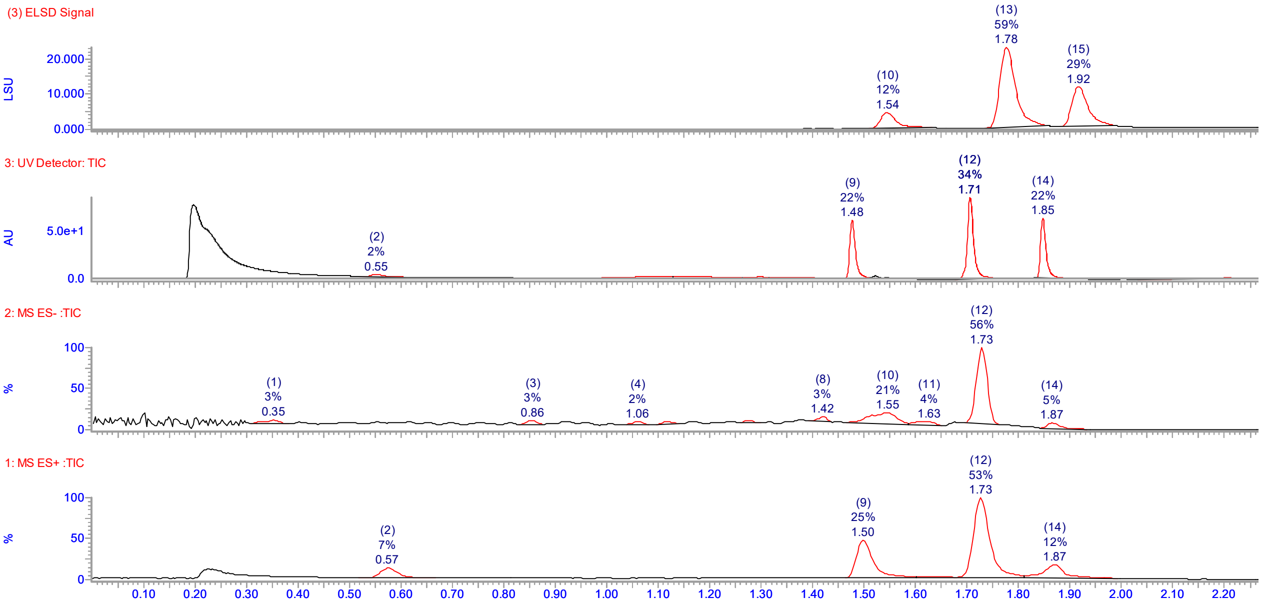


TLC analysis of degraded sample of **1** against carboxylic acid **12**

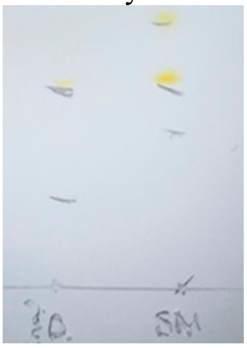

Reaction of **1** with Cysteine

**1** immediately after addition of cysteine

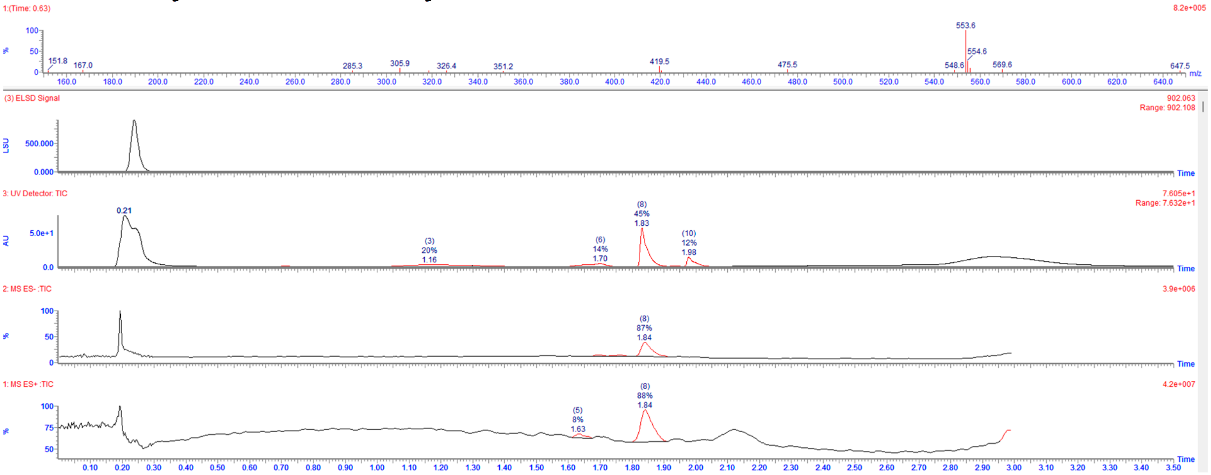


**1** 1 Hour after addition of cysteine

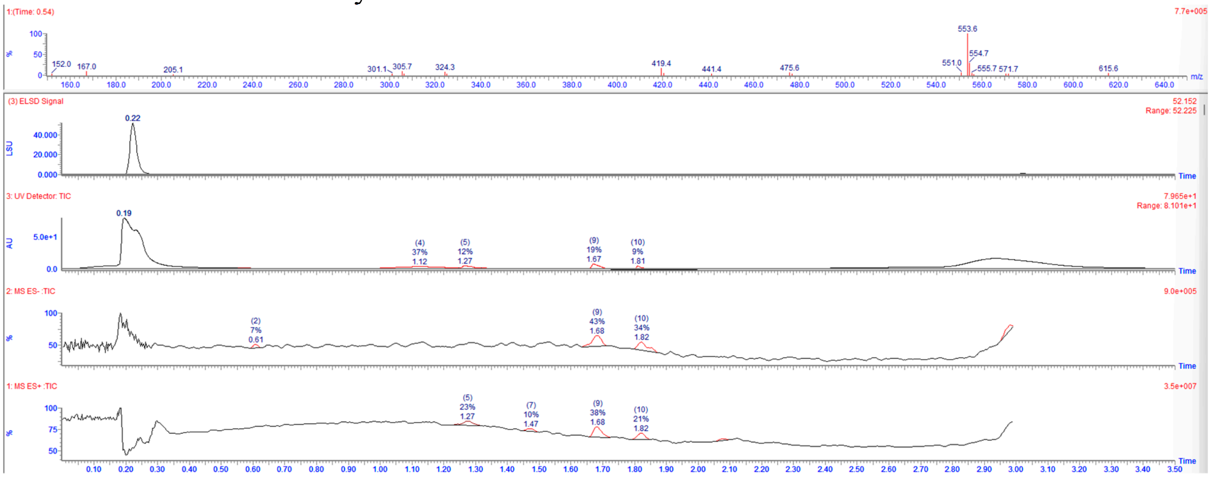


**1** 72 Hours after addition of cysteine

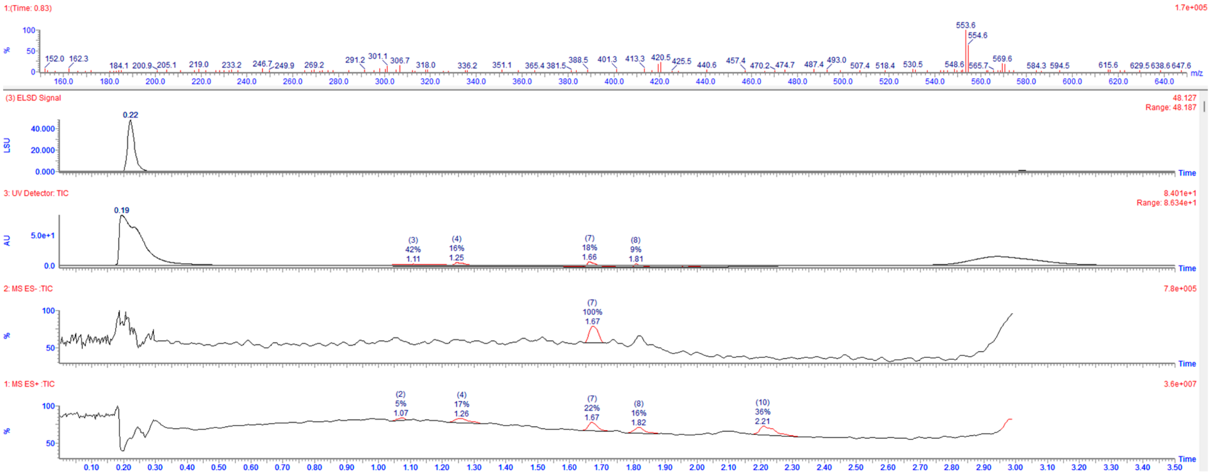

**NMR stability studies**

$^{1}H$ and $^{19}F$ NMR analysis of a sample of **1** in DMSO was taken immediately and periodically at 2, 3, 4 and 5 days. Sample was kept in NMR tube at room temperature during periods between analysis. Relative integrations of **1** and degradation products were plotted against time for both 1H and 19F spectra, which showed the half-life under these conditions to be around 2.5 days.

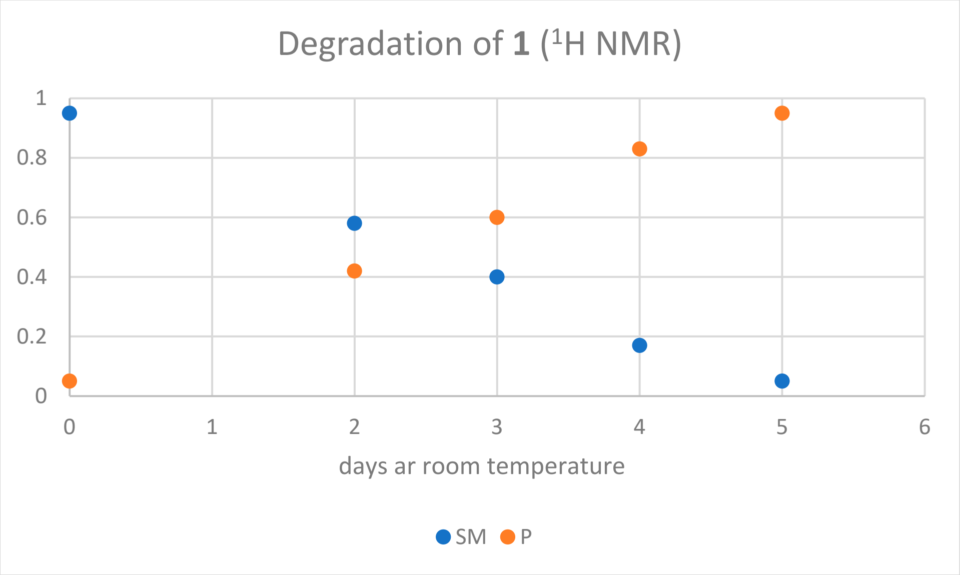


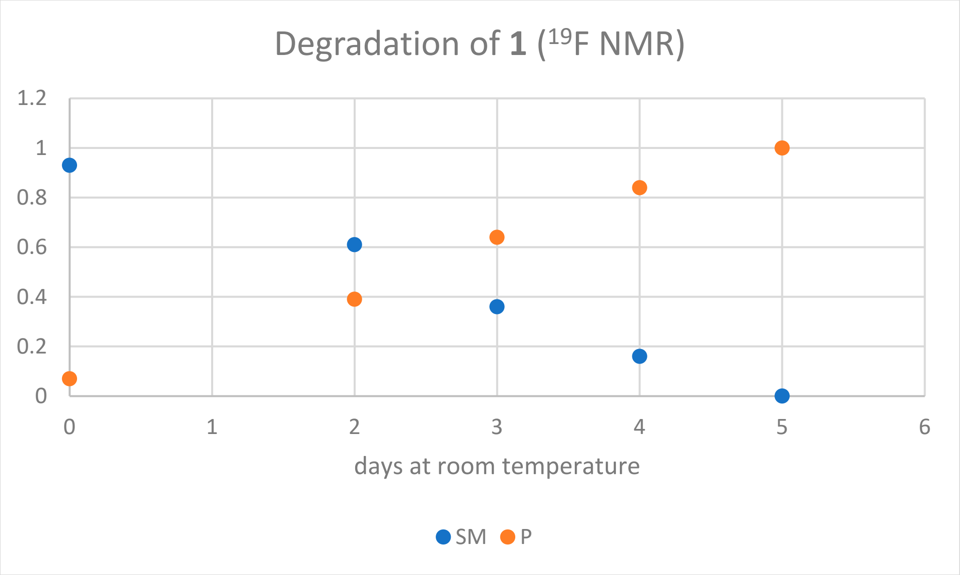

## Biological Assays

### Cell homogenisation

Cultured HEK293 cells were grown until 80-90% confluency, collected, and washed twice in PBS. Collected cell pellets were resuspended in 0.2 M sucrose solution and freeze-thawed three times before being homogenised using a syringe and 25 G needle. Lysed cells were centrifuged at 17,000 g for 30 min at 4°C. The supernatant was collected, and protein quantified using Bradford reagent.

### Acid ceramidase activity assay

To measure acid ceramidase activity, an assay previously described by Bedia et al., (2010) was miniaturised to a 384-well format. The enzymatic assay was carried out in black flat bottom 384-well plates. Briefly, if using cell lysate, each well contained 7.45 µL assay buffer (25 mM sodium acetate pH 4.5) and 0.05 µL 4 mM RBM14-C12 (Avanti, USA) in ethanol (20 µM final substrate concentration), 2 µg protein in 2.4 µL 0.2 M sucrose, and 0.1 µL compound (control wells contained 0.1 µL DMSO). If using purified acid ceramidase protein (Peak Proteins), the experimental set up was similar, but 100 nM protein was used, and the assay buffer contained 25 mM sodium acetate pH 4.5, 0.1% Pluronic F-127, 0.5 mM EGTA, 0.05% BSA, 1 mM TCEP. Blank wells contained the same reaction mixes but without the protein samples. The reaction started upon addition of the protein sample. The plate was incubated at 37°C for 3 h. The reaction was stopped upon addition of 10 µL fresh detection mix (2.5 mg/mL $NaIO_4$ in 100 mM glycine/NaOH buffer pH 10.6) and the plate incubated for a further 1.5 h in the dark. Fluorescence was measured using a microplate reader (PHERAstar FSX; BMG Labtech) at excitation and emission wavelengths of 350 nm and 450 nm respectively.

### Preincubation Acid ceramidase activity assay

The protein was incubated with the compound, at the $IC_{50}$ concentration, at 37°C for the following time points: 0h, 1h and 3h. For each time point a DMSO control was also run. After the preincubation was complete the assay was run as described above in the acid ceramidase activity assay. In each well, 2 µg of preincubated protein was added. The incubation time of the activity assay was reduced from 3h to 0.5h but all other parameters remained the same.
